# Bilateral mode exoskeleton for hand rehabilitation with wireless control using 3D printing technology based on IMU sensor

**DOI:** 10.1016/j.ohx.2023.e00432

**Published:** 2023-06-02

**Authors:** Triwiyanto Triwiyanto, Sari Luthfiyah, I. Putu Alit Pawana, Abdussalam Ali Ahmed, Alcham Andrian

**Affiliations:** a Department of Medical Electronics Technology, Poltekkes Kemenkes Surabaya, Indonesia; b Intelligent Medical Rehabilitation Devices Research Group, Department of Medical Electronics Technology, Poltekkes Kemenkes Surabaya, Indonesia; c Faculty of Medicine, Physical Medicine and Rehabilitation, Universitas Airlangga, Indonesia; d Department of Mechanical and Industrial Engineering, Bani Waleed University, Libya

**Keywords:** Exoskeleton, Bilateral rehabilitation, Wireless control, Post-stroke, *Microcontroller*

## Abstract

This research aimed to develop an open-source exoskeleton for hand rehabilitation (EHR) device that can be controlled wirelessly in bilateral mode. This design has the advantage of being light and being controlled easily using WiFi-based wireless communication by non-paretic hands. This open-source EHR composed of two parts, namely the master and slave parts, each of which uses a mini ESP32 microcontroller, IMU sensor, and 3D printing. The mean RMSE obtained for all exoskeleton fingers is 9.04°. Since the EHR design is open source, the researchers can create and develop rehabilitation device for the therapeutic process of patients who are paralyzed or partially paralyzed independently using healthy hand.

## Graphical abstract

The proposed hardware exoskeleton for hand rehabilitation. The diagram block of the Hand exoskeleton model with bilateral rehabilitation mode.

**Figure:**
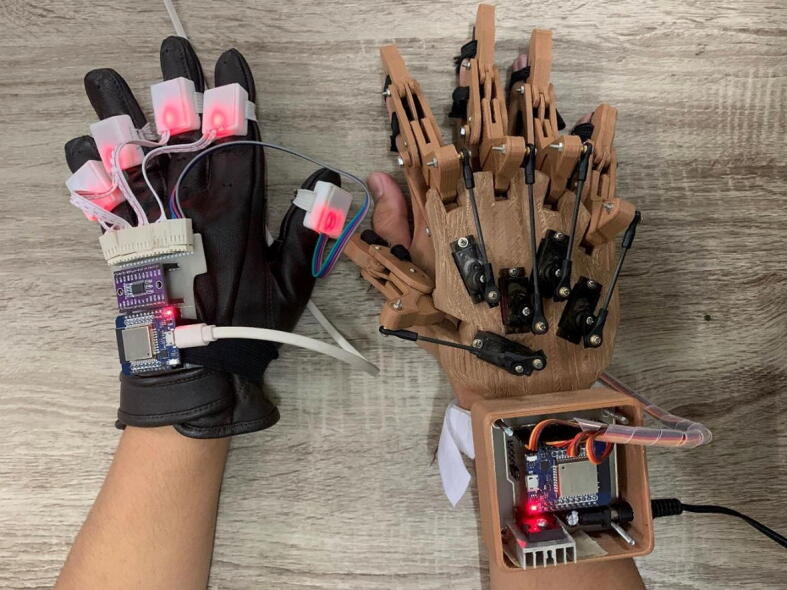

**Specifications table**

**Table:** 

Hardware name	*Exoskeleton for Hand Rehabilitation (EHR) with Wireless Remote Control*
Subject area	• *Electronics and Microcontroller system* • *Rehabilitation Engineering*
Hardware type	• Measuring physical properties and in-lab sensors • Field measurements and sensors • Electrical engineering and computer science • Mechanical engineering and materials science • *Mechatronic engineering*
Closest commercial analog	*This hardware provide wireless control from another healthy hand*
Open source license	https://creativecommons.org/licenses/by-sa/4.0/
Cost of hardware	51.9 *US$*
Source file repository	DOI: https://doi.org/10.17605/OSF.IO/7AGPU
OSHWA certification UID	ID000010

Hardware in context

As wearable robotics develops, exoskeletons are produced and created to support and strengthen lower or upper limbs to carry out various duties [1]. Exoskeletons are utilized in medicine to decrease rehabilitation costs and help patients with neurological illnesses. An exoskeleton design must be adaptable to the user's needs in terms of model and size. The utilization of 3D printing enables us to create and manufacture components with complex forms and configurations [2]. The 3D printed exoskeletons could potentially assist patients in their recovery at home, even without the need for direct supervision from medical professionals such as doctors or nurses [3]. Furthermore, patient who is suffering from hemiplegic stroke, experiences paralysis in one of his limbs so (s)he can use his other limb to carry out activities. In this case, when rehabilitation is not carried out on the paralyzed hand, it can result in loss of muscle mass or atrophy [4], [5], [6]. Several previous researchers have proposed exoskeleton rehabilitations models, especially for hands. In this case, Loconsole et al. developed a hand exoskeleton (BRAVO hand exoskeleton) using a bilateral rehabilitation mode so that post-stroke patients can rehab independently [7]. However, their study used EMG signals to control the hand exoskeleton device, where it requires pre-processing and pattern recognition before used to control the exoskeleton device. The study built the exoskeleton design using a metal frame and a DC motor, obtaining a total weight of 1 kg. On the other hand, another researcher, Powe Heo, developed a power assistive hand exoskeleton (OFX) using a pneumatic cylinder [8]. In his study, the hand exoskeleton made can only move the index finger and thumb. Furthermore, other researchers have developed a hand exoskeleton using 3D printing technology in order to reduce the device’s weight. One of the researchers is Ho et al., who developed a hand exoskeleton device for post-stroke patient training using 3D printing technology and five linear actuators [9].

The open and close hand exoskeleton movements were controlled based on EMG signal activity. In this case, Sergio developed a hand exoskeleton (five fingers) using 3D printing design [10]. Several small servo motors drove the hand exoskeleton to obtain a lightweight and small size device. However, their research focused on 3D design, so no information concerning the control system is provided. Furthermore, Badesa at al. [11] has carried out a development of exoskeleton controlled by a non-paretic hand. In their study, the hand exoskeleton on the paretic hand was controlled through a non-paretic hand wearing 5DT (Five Dimension Technology) connected via a cable [12], [13], [14]. Based on advancements in hardware design and technology used in earlier studies, various things need to be developed. One of the developments needed is a hand exoskeleton with a wireless-based control system that was proposed in this study. The use of wireless communication in this design facilitates the hand exoskeleton control process and minimize cabling. In addition, the use of IMU sensor for controlling does not require signal processing. Furthermore, the hardware designed in this study is an exoskeleton for hand rehabilitation (EHR) which allows the user to carry out rehabilitation independently using the non-paretic hand (Fig. 1), hereinafter referred to as bilateral rehabilitation. This EHR can be controlled wirelessly using gloves through via WiFi based on the ESP-NOW function. In addition, the EHR was designed using 3D printing, micro servo motors, and ESP32 mini microcontrollers to obtain a light and simple EHR.

**Fig. 1:**
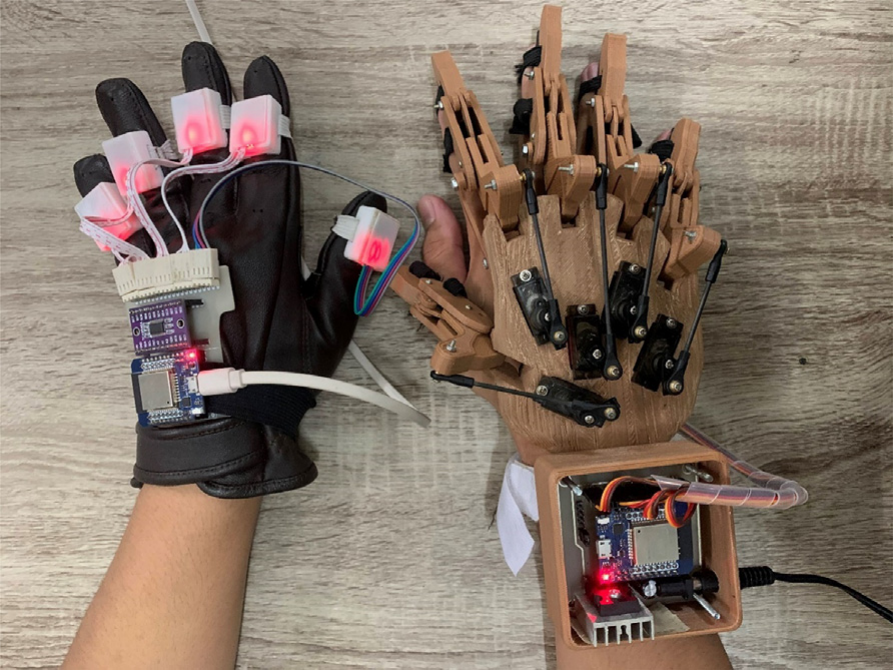
The proposed hardware of the exoskeleton for hand rehabilitation.

## Hardware description

**Fig. 2:**
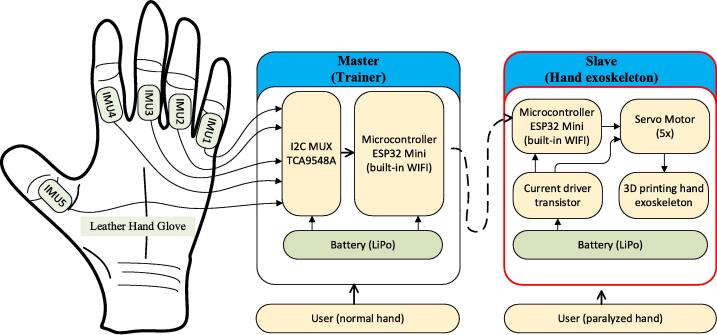
The diagram block of the Hand exoskeleton model with bilateral rehabilitation mode.

Exoskeleton for hand rehabilitation (EHR) generally focuses on developing 3D printing design models, selecting the suitable actuators (motor DC [15], [16], [17], [18], servo motor [19], the linear actuator [20] or pneumatic system [21]), and pattern recognition of the EMG signal used as a control signal. However, for the most part, the hardware proposed earlier is not an open-source hardware. In this case, EHR hardware consists of two parts, master and slave, as shown in Fig. 2. The master block consists of a microcontroller ESP32 mini Wemos D1, battery, and leather hand glove with five IMU sensors.

Five IMU sensors are used to detect the movement of the glove when moved by fingers. The output of the IMU sensor is then connected to IC TCA9548A, which is an I2C multiplexer. The microcontroller further choose the IMU sensor to measure in turn. The results of the angle measurements taken by the five IMU sensors are then sent to the slave circuit using ESP-now communication.

Furthermore, the master part is applied to the non-paretic hand. Meanwhile, the other part, the slave block, consists of the ESP32 mini Wemos microcontroller, driver transistor for high current power supply, battery, five servo motors, circuit box, and hand exoskeleton 3D printing. The ESP32 microcontroller in the slave circuit then commands five servo motors to follow the movement according to the angle received by the five IMU sensors. Current driver transistor in the slave circuit is used to provide voltage to the microcontroller and servo motor with sufficient current. The slave part is used on paralyzed hand due to post-stroke.

### Exoskeleton hardware

**Fig. 3:**
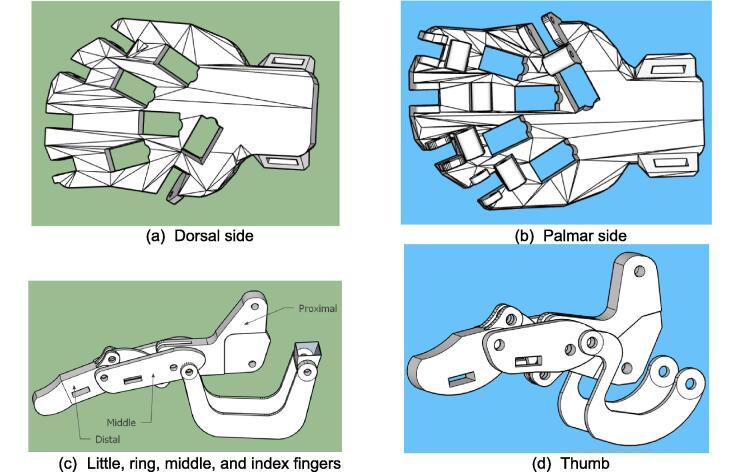
Design of exoskeleton for hand rehabilitation using SketchUp application.

**Fig. 4:**
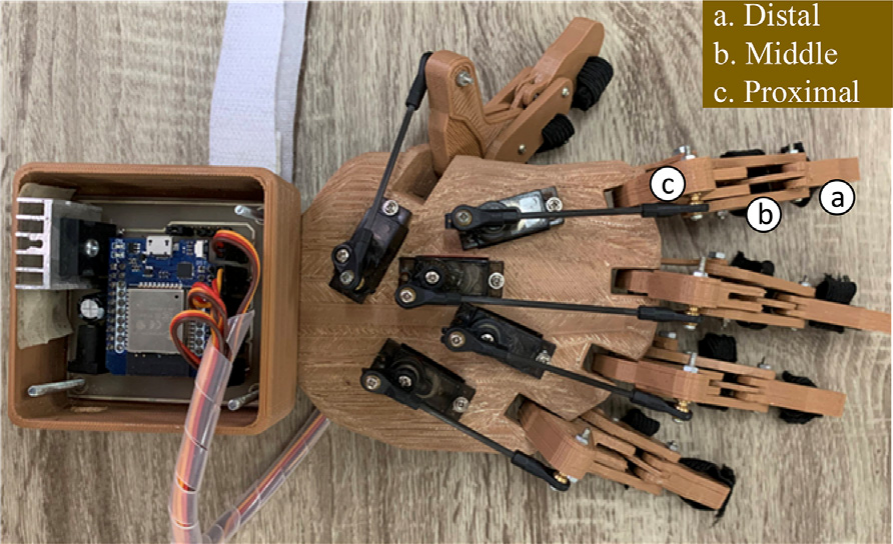
The 3D printed of exoskeleton for hand rehabilitation which consists of 3D printing materials, servo motor, and microcontroller ESP32 Wemos D1.

The exoskeleton for Hand Rehabilitation (EHR) was designed using the 3D design application program Sketchup (SketchUp Pro 2019, Version 19.0.685 64-bit, 2018 Trimble Inc., Colorado, US), as shown in Fig. 3. It consists of three main parts, namely the dorsal side (Fig. 3(a)), palmar side (Fig. 3(b)) and fingers (Fig. 3(c) and Fig. 3(d)). In this case, the dorsal side is used as a holder for five servo motors that move the little, ring, middle, index, and thumb fingers. Each finger consists of three main phalanges: distal, middle, and proximal phalanges (Fig. 3(c) and Fig. 3(d)) [22], [23]. Distal and proximal are connected via a link. Meanwhile, the five proximal phalanges are attached to the palms. Each proximal phalanx is connected to each servo motor via a rod so that when the servo motor rotates CW 0-90°, the proximal phalanx will be pushed, and vice versa; when the servo motor rotates CCW 90°, the proximal phalanges are retracted. The EHR was designed based on the dimensions of an adult male hand with palm dimensions of 150 mm × 92 mm × 9 mm (L × W × H). EHR is a hardware integration consisting of mechanical and electronic systems, both master and slave, as shown in Fig. 4.

### Hardware circuit

**Fig. 5:**
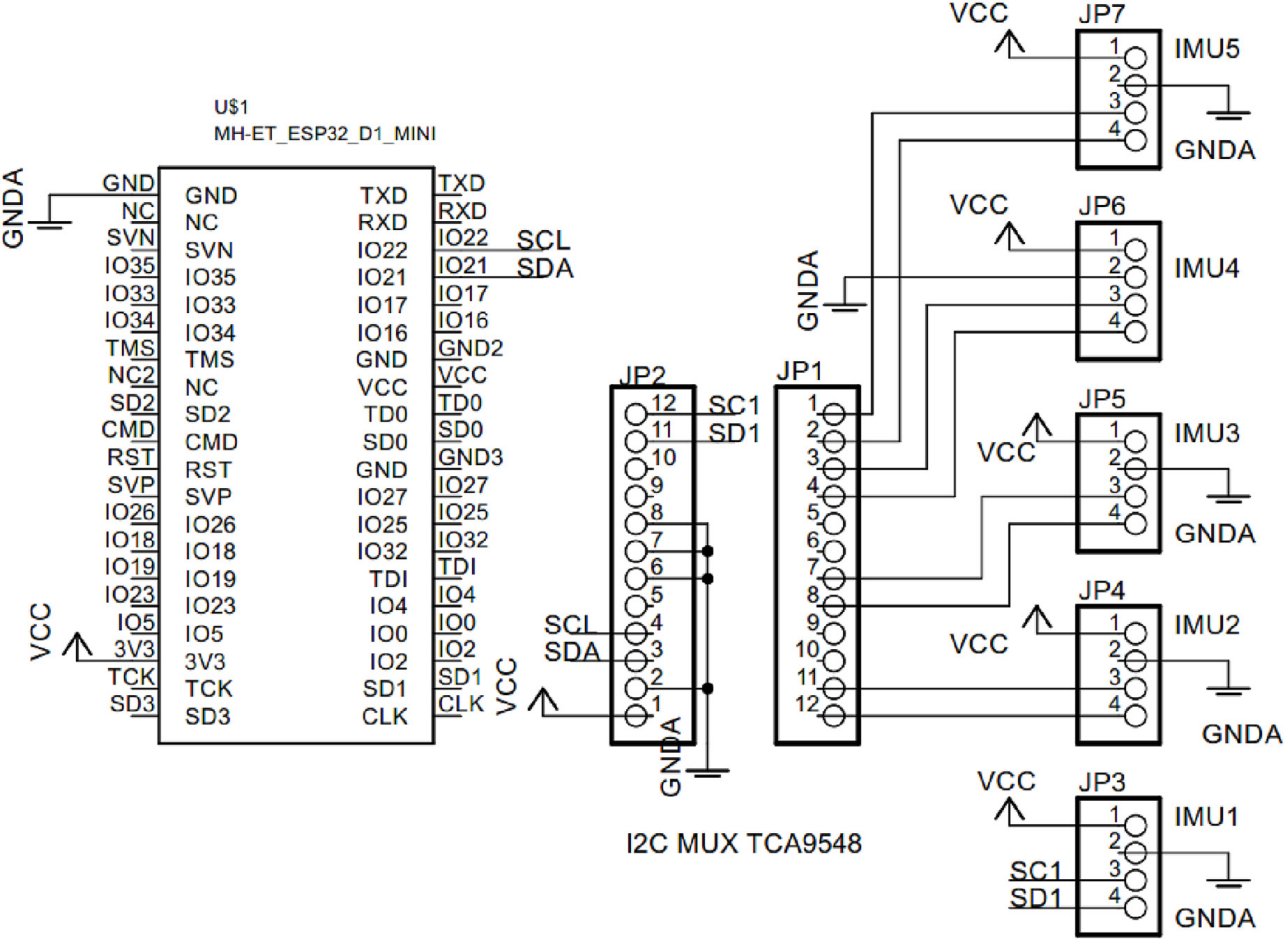
Master hardware circuit.

**Fig. 6:**
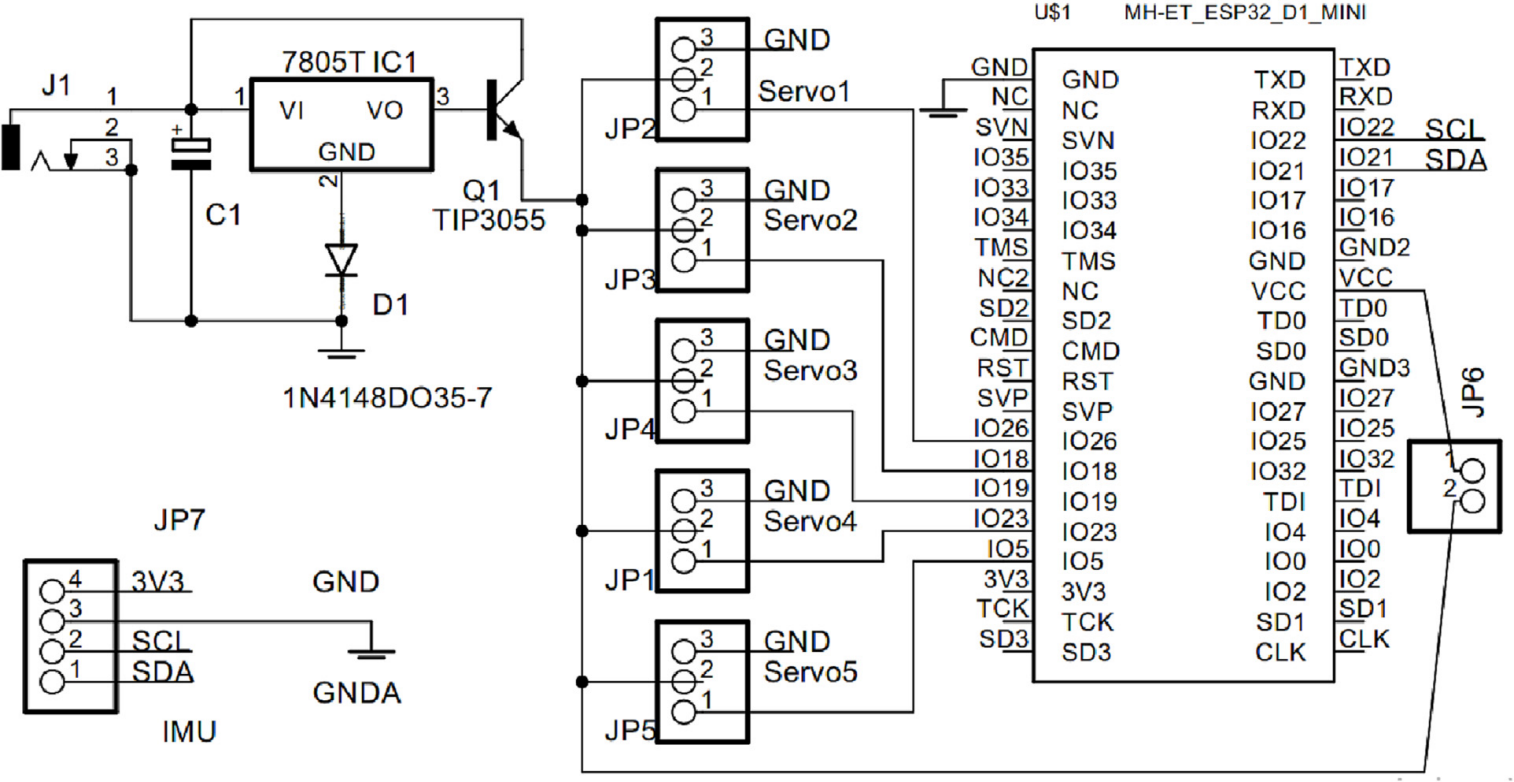
Slave hardware circuit.

The hardware circuit consists of two main parts, master and slave, as shown in Fig. 5 and Fig. 6. In addition, it also consists of an ESP32 microcontroller [24], [25]. In this study, the ESP32 is a built-in mini Wemos D1 which is powered by Xtensa® dual-core 32-bit LX6 microprocessor(s) with specification as explained in the Table 1. In the master section, the hardware is composed of a microcontroller ESP32 mini Wemos D1, I2C multiplexer TCA 9548, and five IMU sensors. The IMU sensor is based on the GY-521 module composed of MPU6050. The I2C pin, GPIO21, and GPIO22 are connected to the SCL and SDA input of TCA9548. Meanwhile, the I2C multiplexer TCA9548 communicates to the five IMU sensors alternately.

**Table 1:** Summary of ESP32 Specification.

Items	Specification
MCU	Xtensa Dual Core 32-bit LX6, 600 DMIPS
802.11b/g/n Wi-Fi	Yes, HT40
Bluetooth	Bluetooth 4.2 and below
Typical Frequency	160 MHz
SRAM	512 kBytes
Flash	SPI Flash up to 16 MBytes
GPIO	36
Hardware/ Software PWM	1/ 16 channels
SPI/ I2C/ I2S/ UART	4/2/2/2
ADC	12-bit
CAN	1
Ethernet MAC Interface	1
Touch Sensor	1
Temperature Sensor	YES
Working Temperature	−40 °C −12 °C

The five IMU sensors are placed on the hand glove made from leather material. The IMU sensors (GY-521 module) are located on the little, ring, middle, index, and thumb fingers. The angle reading resulting from the IMU sensor is then sent to the slave hardware wirelessly (WIFI protocol) using the ESP-NOW function. The slave hardware circuit consists of the ESP32 mini Wemos D1 microcontroller, five servo motors, TIP 3055 transistor driver, LM7805, 1000 uF/25 V capacitor, LiPo Battery, and 1 N4001 diode. All electronic components of the slave circuit are soldered to the printed circuit board (PCB) with a PCB size of 65 mm × 65 mm.

The power supply circuit consists of high-power transistor TIP3055, 7805, diode 1 N4001, capacitor 1000uF, jack DC black connector, and battery (Fig. 6). According to the servo motor specification, motor servo requires an input voltage of 4.8 V − 6.0 V and operating current (5.0 V) of ∼ 2.7 mA (idle), ∼70 mA (no load), ∼400 mA (Stall). Therefore, the minimum required current to drive five servo motors is 2,000 mA. TIP3055 is able to drive the supply current until 15A; therefore, it will be able to provide the required current for the servo motors. In the slave hardware, we alternately put the IMU sensor on one of the five fingers to validate the angle between the master and the slave.

### EHR firmware

**Fig. 7:**
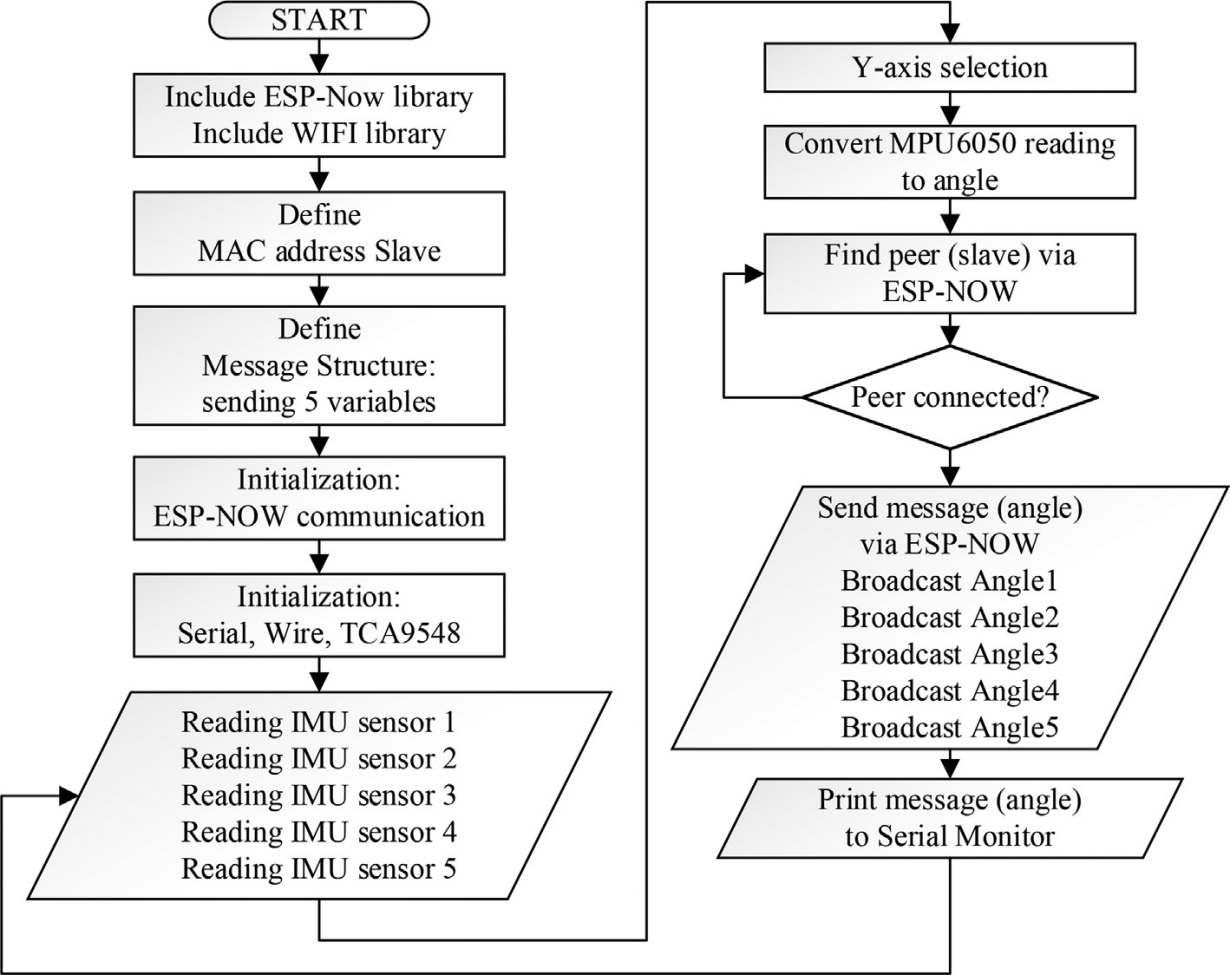
Firmware flowchart of EHR for master.

Both firmware (master and slave) was developed in this EHR development using Arduino software (Version 1.8.4, Arduino LLC, New York, US). The required libraries to communicate the master and slave are esp_now and WiFi. Furthermore, the slave required the ESP32Servo library to control the motor servo. Further details for the master firmware (Fig. 7) are explained as follows; the slave's (MAC) address must be determined so that the master part is able to communicate with the slave part. First, the Arduino MACaddress.ino program must be run into the slave microcontroller to get the address. In this study, the slave MAC addresses for this hardware device are 0xEC, 0x94, 0xCB, 0x64, 0xBC, and 0x84 (these addresses may differ for each microcontroller). In the master firmware, the message structure is defined in five variables to include data from the five IMU sensors. The IMU sensor produces measurement results from a three-axis accelerometer (x, y, and z) and a three-axis gyroscope (x, y, z), but in this study, the measurement from the accelerometer y-axis was chosen for control purposes. This is because the finger flexion and extension movements are linear to the y-axis accelerometer output. Furthermore, the output of the × and z axis accelerometer does not produce a significant value to changes in the flexion and extension movements of the fingers. The MPU6050 chip (GY-521 module) displays accelerometer values in the range of −17000 to 17000. Therefore, the range must be converted into angle units (°) using the MAP function on Arduino.

**Fig. 8:**
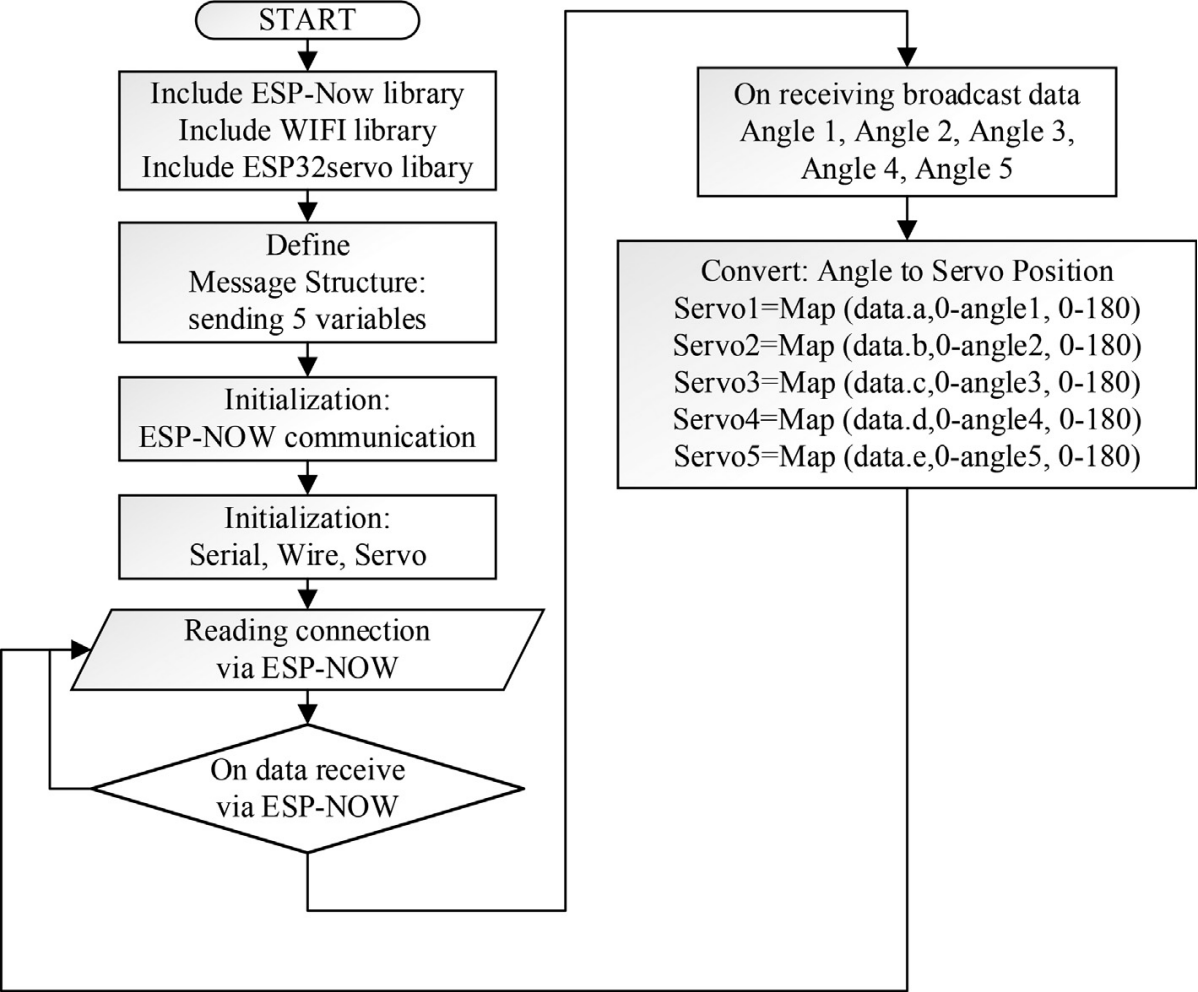
Firmware flowchart of EHR for slave.

The address of the slave is determined before the master broadcasts the data packets. It was carried out to synchronize the slave. In both master and slave firmware, some libraries are declared in the initialization stage to recognize some functions. Apart from broadcasting the measurement data from the IMU sensor to the slave, the master also outputs data to the computer monitor via the serial monitor function to validate the output. In this case, the program will run continuously according to the loop in order to run the system in real-time, as shown in Fig. 7, until the power supply is turned off. Likewise, the slave firmware flowchart shown in Fig. 8 can be explained as follows; specify several libraries, including esp_now, ES32Servo, and WiFi. The slave firmware will receive the data broadcast (angle1, angle2, angle3, angle4, and angle5) from the master. The angle range is then converted to the servo angle range (0–90). In addition, a map function is required to convert the range from 0 to 90 (angle) to 0–90 (servo). After receiving the data, the variable is printed serially and forwarded to control the servo motor. Thus, the program repeats forever to detect broadcast data and control the servo motor, as shown in Fig. 8.

### Easy to control the EHR

EHR is used to rehabilitate post-stroke hemiplegic patients, namely patients who experience paralysis of one of his limbs so that he can still use the other limb [3], [23]. This EHR system was designed using 3D printing design, a microcontroller, five mini servo motors, IMU sensors, and wireless communication based on the ESP-NOW function. The resulting product is a lightweight and simple open-source EHR. Weight comparison of the hand exoskeleton design with other studies is shown in Table 2. Furthermore, when EHR is used on the paretic hand, the non-paretic hand can control it wirelessly through a hand glove with an IMU sensor attached to the finger glove.

**Table 2:** The weight comparison among other studies.

**Design**	**Hand Glove/Master** **(kg)**	**Actuator/Slave** **(kg)**
[14]	0.258	2.200
[21]	0.220	16.000
Proposed design	0.105	0.335

### Cost

**Fig. 9:**
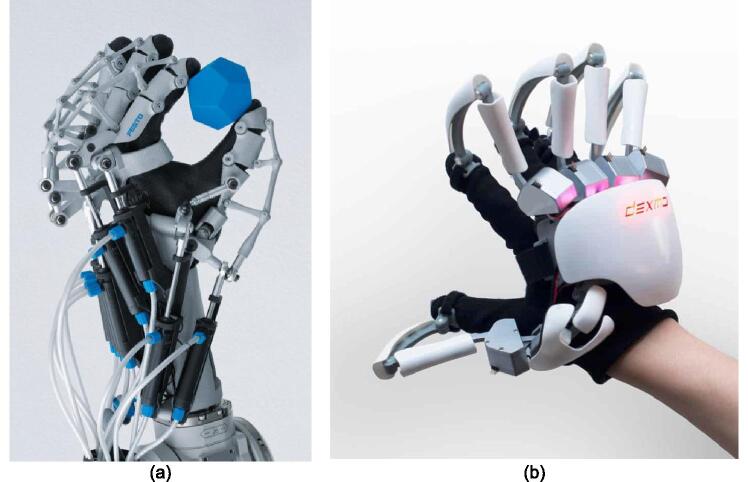
(a) exohand festo [26], (b) dextahand [27].

The expensive price of commercial exoskeleton products [26], [27] on the market (>1000 USD) as well as the long and expensive rehabilitation process become the obstacles in carrying out post-stroke rehabilitation, especially for people who have middle to low incomes (Fig. 9). However, this design has a low cost, namely 51.9 US $ for one EHR product. In addition, this design is open source so that other researchers can develop it with other features and improvement.

### Summary

EHR can be used for independent rehabilitation process for paralyzed hand using the non-paralyzed hand and wireless means. The contribution of this research is explained as follows:

(a) The hardware of this design is simple and portable because it uses a mini ESP32 with a minimum system board dimension of 39 mm × 31 mm so that this design has a wearable system.
(b) The hand exoskeleton (EHR) device can be controlled wirelessly using WiFi via the esp_now function so that no cables are required to control the hand exoskeleton device.
(c) The hand exoskeleton (EHR) device can be controlled using gloves that have an IMU sensor attached to each finger, so it does not require pre-processing and pattern recognition for control purposes.
(d) Each finger exoskeleton on the hand can be controlled individually using the finger on the glove.

## Design files summary

### Design file

This section describes the resulting design files, both the hardware design (schematic and printed circuit board (PCB)) and the firmware for operating the hand exoskeleton, as shown in Table 3. The firmware for detecting the slave microcontroller address is also presented in Table 3. Furthermore, the mechanical design of hand exoskeletons using 3D printing materials is also attached to this study (see Table 4).

**Table 3:** Design file summary of exoskeleton for hand rehabilitation.

**Design file name**	**File type**	**Open source license**	**Location of the file**
*Slave.sch*	schematic, eagle file	CC BY-SA 4.0	DOI: 10.17605/OSF.IO/7AGPU
Slave.brd	board, eagle file	CC BY-SA 4.0	DOI: 10.17605/OSF.IO/7AGPU
Master.sch	schematic, eagle file	CC BY-SA 4.0	DOI: 10.17605/OSF.IO/7AGPU
Master.brd	board, eagle file	CC BY-SA 4.0	DOI: https://doi.org/10.17605/OSF.IO/7AGPU
MacAdress.ino	Arduino	CC BY-SA 4.0	DOI: 10.17605/OSF.IO/7AGPU
Exo-master.ino	firmware, Arduino	CC BY-SA 4.0	DOI: 10.17605/OSF.IO/7AGPU
Exo-slave.ino	firmware, Arduino	CC BY-SA 4.0	DOI: 10.17605/OSF.IO/7AGPU
Hand exoskeleton.skp	3D printing source, sketchup file	CC BY-SA 4.0	DOI: 10.17605/OSF.IO/7AGPU
EHR-step.rar	3D printing source, *.step (FreeCad)	CC BY-SA 4.0	DOI: 10.17605/OSF.IO/7AGPU

**Table 4:** Bill of materials of a hand exoskeleton.

**Designator**	**Component**	**Number**	**Cost per unit (USD)**	**Total cost** **(USD)**	**Source of materials**	**Material type**
U1, U2	ESP32 D1 wemos board	2	4.3200	8.6400	https://www.aliexpress.com/item/1005004005628679.html	Semi-conductor
S1, S2, S3, S4, S5	MG90S Metal gear Digital 9 g Servo	5	1.5500	7.7500	https://www.aliexpress.com/item/1005004203611301.html	Plastic, Metal, and semi-conductor
	Metal Servo Steering Rod 28 T	5	2.2000	11.0000	https://www.aliexpress.com/item/1005003848853409.html	Metal and plastic
P1, P2, P3, P4	Variable resistor 10 k	4	0.1760	0.7040	https://www.aliexpress.com/item/32844752123.html	Metal and ceramic
SW1	Switch push button	1	0.1750	0.1750	https://www.aliexpress.com/item/10000275217379.html	Plastic, metal
3D printing	PLA Filament 1.75 mm Metal Silk PLA 3D Printer Filament	1	33.9000	33.9000	https://www.aliexpress.com/item/4001230078114.html	Polylactic Acid
Q1	TIP3055 3055 TO-247 15A 100 V	1	0.2560	0.5120	https://www.aliexpress.com/item/33004236891.html	Semi-conductor
IC1	7805	1	0.0880	0.0880	https://www.aliexpress.com/item/1005002784643392.html	Semi-conductor
C1	1000 uF	1	0.0855	0.0855	https://www.aliexpress.com/item/1005002542855250.html	Semi-conductor
J1	Black DC Socket DC Connectors	1	0.0890	0.0890	https://www.aliexpress.com/item/1005002827113327.html	Plastic, metal

### Schematic and board

The exoskeleton hand was designed using the Eagle application program (6.3.0, free version for Windows, CadSoft Computer GmbH, Germany). The schematic file consists of the slave and master. The master circuit consists of a circuit that is directly connected to several servo motors (five servo motors), while the slave circuit consists of a circuit connected to four potentiometers and one switch.

### Firmware

The hand exoskeleton firmware was developed using Arduino application program (Version 1.8.4, visit the website: URL). Table 1 shows two firmware, namely master (Exo-master.ino) and slave (Exo-slave.ino). The master firmware was programmed on the ESP32 mini Wemos D1 (U1) to detect the angular position of the hand glove by IMU sensors (IMU1, IMU2, IMU3, IMU4, and IMU5). Furthermore, the IMU sensor reading results are sent using the esp_now function to the slave. The slave firmware was programmed on the ESP32 mini Wemos D1 (U2) to detect broadcast data from the master microcontroller. When the data are detected, the data would be used to instruct each servo motor to rotate 0–180 depending on the data received.

### 3D printing design

The hand exoskeleton was designed using the SketchUp 2019 application (available in the free version: https://www.sketchup.com/). The hand exoskeleton.skp file shown in Table 3 consists of several parts, including palm and finger. Some things related to 3D printing design can follow the following references [28].

## Bill of materials summary

### Build instructions

#### 3D printing design

**Fig. 10:**
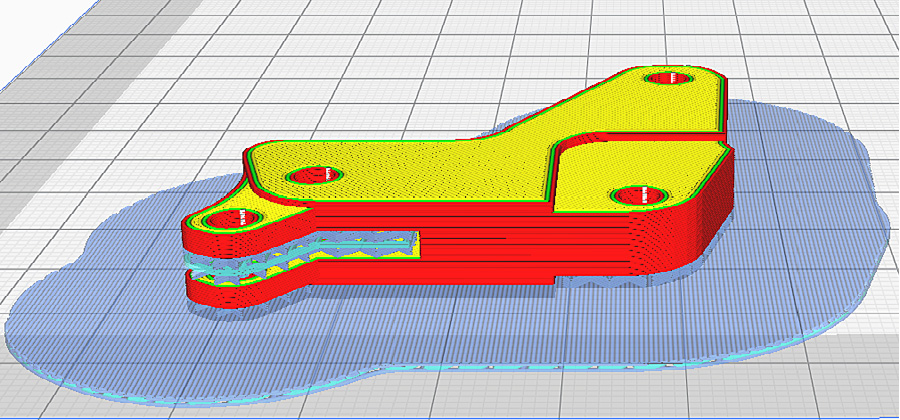
Example of setting to support and build plate adhesion type of raft.

The hand exoskeleton was printed using a 3D printer (Anet, Anet 8 Plus, China) using filament polylactic acid (PLA) material. The printer has X, Y axis position with precision of 0.015 mm, Z axis position with precision of 0.004 mm and layer precision 0.1–0.4 mm in according to Anet 8 plus user manual [29]. In addition, in the 3D printing process, the dimension accuracy of the printed parts is 0.2 mm. The hand exoskeleton design using the Sketchup application (SketchUp Pro 2019, Version 19.0.685 64-bit, 2018 Trimble Inc., Colorado, US) was exported in the STL extension (STL unit is model units and file format is binary). Next, the pre-processing step was carried out, namely by converting the STL to GCODE extension file using the Ultimaker Cura application (Version: 4.10.0, Ultimaker, Utrecht, Netherland) (Fig. 10). The parameter setting in the Ultimaker Cura application was 30% infill. With 30% infill, the density of the 3D printing print is quite dense and strong. The higher the infill value, the higher the density of the printout, including the long print time required. Each section takes a different amount of time, depending on the volume of each file. Furthermore, in the Ultimaker Cura setting, the build plate adhesion (type raft) must be adjusted to bind the object to the hotbed (Table 5). During the printing process, the 3D printer was set with the nozzle temperature parameter setting at 225 °C and the bed temperature at 60 °C, as shown in Table 6 for PLA material (Esun, diameter: 1.75 mm, temperature: 205–225°). In addition, the EHR design was made in adult (Asian) human size. Dimensions of 3D printing parts can be customized by using SketchUp application or setting dimensions through Ultimaker Cura application.

**Table 5:** Setting parameters on Ultimaker Cura.

Parameter	Unit
Profile	Standard quality (0.2 mm)
Nozzle	0.4 mm
Print speed	80%
Infill Density	30%
Infill Line Distance	4.0
Support	Enable
Build plate addesion	Raft extra margin 15 mm

**Table 6:** Setting parameters on 3D printer.

Parameter	Unit
Nozzle temperature	225 °C
Hot bed temperature	60 °C
Print speed	80%

#### Firmware design

**Fig. 11:**
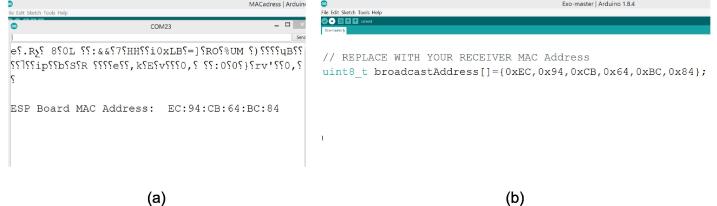
(a) Running Arduino code to obtain the MAC address, (b) placing the MAC address on master program code.

The firmware was designed using Arduino software (Version: 1.8.4). In this case, the MAC address of the slave should be obtained so that the master is able to communicate or broadcast the data to the slave part. In order to get the MAC address, the user should run the MACAddress file into the slave (MacAdress.ino, see Table 1). User will obtain a serial digital number when using an Arduino serial monitor application (for example, EC:94:CB:64:BC:84) (Fig. 11(a)). As a note, the MAC address is different for each microcontroller ESP32. After the MAC address is obtained, the number is inputted into the master program code (see master.ino, in Table 1 and Fig. 11(b).

#### Hardware circuit

**Fig. 12:**
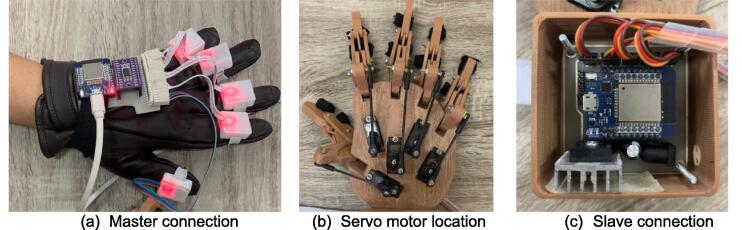
Circuit hardware build instruction.

After the firmware is programmed into each master and slave, the hardware is ready to operate. On the master body, the IMU sensors connectors are connected to the ESP32 mini Wemos D1 header board, as shown in Fig. 12(a). After placing the servo motors at each location on the 3D printing palm, a link rod connecting the servo motor is attached to the finger proximal phalanges of the hand exoskeleton, providing a push and pull movement mechanism (Fig. 12(b)). The servo motor is mounted on the palm using two bolts with holes that are already available during 3D design so that no drilling process is required for assembly. On the slave circuit board, the five servo motors installed on the hand exoskeleton are connected via the supplied cables and connectors, as shown in Fig. 12(c).

The hardware circuit can immediately respond when the power supply is provided. Five IMU sensors are placed in each finger (little, ring, middle, index, thumb fingers), connected to the circuit board, and now ready to control the hand exoskeleton.

## Operation instructions

The power supply must be off before using the EHR device. The EHR is placed on the part of the hand that will receive rehabilitation therapy. The EHR design was made for the treatment of a paralyzed right hand. In this case, each of the elastic straps is put on each hand exoskeleton finger. The left hand can operate the hand exoskeleton by moving the hand in flexion or extension motion. When the EHR device is ready, the power supply is turned on for both the slave and master. Furthermore, the data received by the slave can control the movement of the servo motor connected to the proximal phalanges. Each IMU sensor (5 units) in the master system can control one servo motor.

## Validation and characterization

**Fig. 13:**
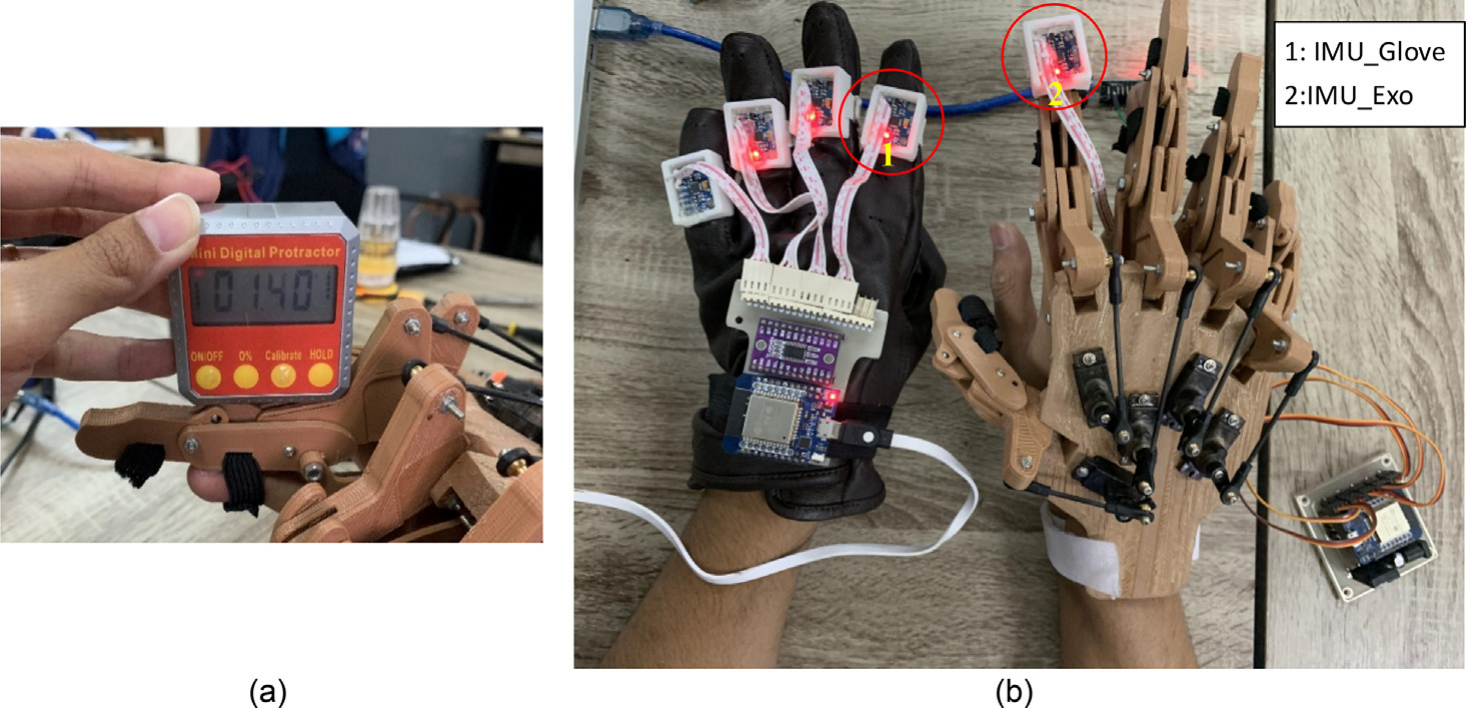
Angle validation and calibration of the hand exoskeleton by using (a) IMU sensor (b) mini digital protractor.

The angular calibration process should be done on the hand exoskeleton before the validation process. The calibration process was carried out by installing a digital angle measuring instrument (Mini Protractor digital, X15-007, China) on the radius of the hand exoskeleton. Then, this process was performed by adjusting certain angles by positioning the servo motor's rotating angle and measuring the angular position of the resulting hand exoskeleton using a digital angle measuring instrument, as shown in Fig. 13 (a). Table 7 shows the results of mean measurements and standard deviations of angles that were performed repeatedly for each setpoint. The mean of standard deviation value obtained for all angular positions measured based on Table 7 is 0.490 ± 0.298%.

**Table 7:** Mean values and standard deviations measure the angle of the hand exoskeleton for several angular positions using digital angle-measuring instruments. The measurement process for each angle was repeated ten times for each respondent (10 respondents).

Setpoint Angle	Measured angle	
(°)	Mean (°)	SD. (°)
0	0.571	0.33
20	19.87	0.298
30	29.89	0.84
60	60.17	0.509
80	80.14	0.237
90	89.59	0.324

**Fig. 14:**
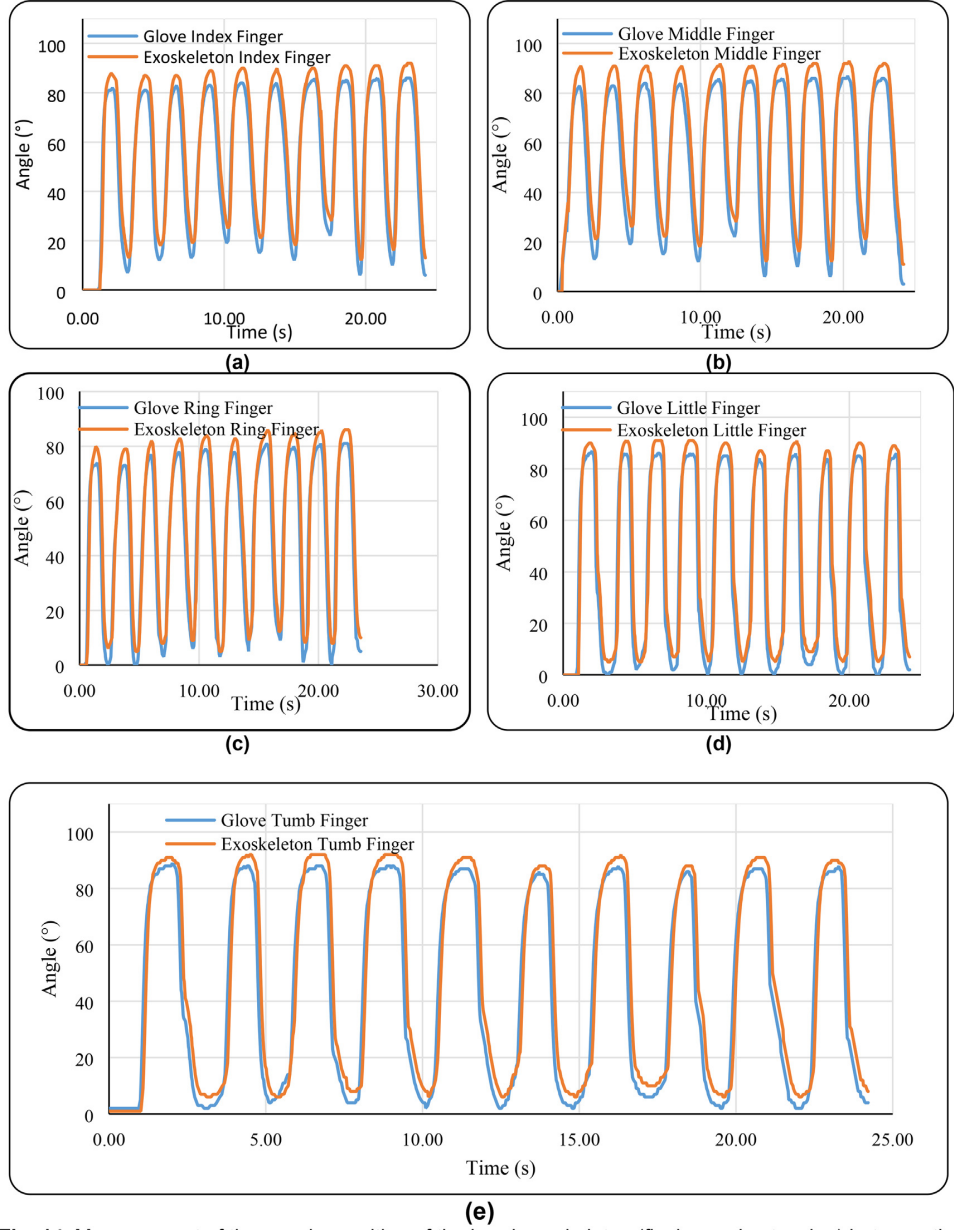
Measurement of the angular position of the hand exoskeleton (flexion and extension) between the sensor IMU (master) and the fingers exoskeleton (slave) on (a) index, (b) middle, (c) ring, (d) little, and (e) thumb fingers.

Furthermore, the angle of motion of the exoskeleton of the hand was validated using the IMU sensor by placing one of the IMU sensors on one of the exoskeleton fingers, as shown in Fig. 13 (b) alternately. The validation process started from the index, middle, ring, little finger, and thumb. Once the master and slave hardware system operates, the finger on the glove can make periodic flexion and extension movements. In order that the flexion and extension movements carried out by the hand glove can run periodically; so the current study used a metronome application. A metronome application (TempoPerfect Metronome Software, NCH Software, Australia, URL: homepage) was used in this study to guide the flexion and extension movements which can be repeated periodically and permanently. In this test, the metronome was set at 20 bpm (beats per minute or cycles per minute). In this case, Fig. 14 shows the IMU sensor (angular position) between the glove and the exoskeleton of the hand. Compared to glove movements, the performance measurement of exoskeleton hand movements was calculated using the root mean square error (RMSE) as shown in Eq. (1).

(1) $$\mathrm{RMSE}=\sqrt{\frac{\sum_{i=1}^{N}{\left(y_{i}-x_{i}\right)}^{2}}{N}}$$

where yi indicates the predicted values, xi shows the actual values, and N is the measurement data. The result of the measurement of the average RMSE value for all exoskeleton fingers is 9.04° (Table 8).

**Table 8:** The results of the root mean square error (RMSE) value measurement on each finger through a comparison between the measurement results of the angle of the fingers on the hand glove and the hand exoskeleton.

**Finger**	**RMSE (°)**
Index	8.89
Middle	7.13
Ring	8.89
Little	11.07
Thumb	9.23

## Conclusion

This study presents the development of an open-source exoskeleton for hand rehabilitation (EHR) device that can be controlled wirelessly in bilateral mode. This design requires a low cost, namely 51.9 US $ for one EHR product. The mean RMSE and standard deviation measured based on the hand glove and hand exoskeleton is 9.04 ± 1.40°. In the future work, researchers can integrate several types of sensors to control the exoskeleton of the hand such as IMU sensor, EMG senso,r and force sensor. The EMG sensor can be used to detect the muscle activities and force sensor can be used to measure the grasp force.

**Ethics statements**

The author confirmed that informed consent was obtained from the subjects. This research has passed the ethical examination conducted by Health Research Ethics Committee Poltekkes Kemenkes Surabaya, Indonesia, No.EA/1245/KEPK-Poltekkes_Sby/V/2022.

## CRediT authorship contribution statement

**Triwiyanto Triwiyanto:** Conceptualization, Methodology, Software. **Sari Luthfiyah:** . **I. Putu Alit Pawana:** Validation. **Abdussalam Ali Ahmed:** . **Alcham Andrian:** .

## Declaration of Competing Interest

The authors declare that they have no known competing financial interests or personal relationships that could have appeared to influence the work reported in this paper.
